# Episodic Memory in Amnestic Mild Cognitive Impairment at Risk for Alzheimer’s Disease: Spanish Validation of the TYM-MCI

**DOI:** 10.3390/jcm15031236

**Published:** 2026-02-04

**Authors:** Ámbar Belmar-Moreno, Felipe Egaña-García, Amparo Castillo-Borredá, Erika Caballero-Muñoz, Vicente Gatica-Elgart, Fernando A. Crespo, Paula Salinas-Lainez, Norma Muñoz-Ojeda, Danton Freire-Flores, Claudia Carvallo-Varas, Héctor Burgos

**Affiliations:** 1 Escuela de Psicología, Facultad de Ciencias Sociales, Universidad Mayor, Av. Manuel Montt 367, Santiago 757008, Chile; flga.ambarbelmar.neurop@gmail.com (Á.B.-M.); ps.egana@gmail.com (F.E.-G.); psi.vicente.ge@gmail.com (V.G.-E.); 2 Facultad de Ciencia de la Salud, Universidad del Alba (UDALBA), Av. Ejército Libertador 171, Santiago 8370007, Chile; 3 Escuela de Psicología, Facultad de Ciencias Sociales y Comunicaciones, Universidad Santo Tomás, Av. Ejército Libertador 171, Santiago 8370003, Chile; amparocastillobo@santotomas.cl; 4 Escuela de Enfermería, Facultad de Salud, Universidad Santo Tomás, Santiago 8370003, Chile; ecaballerom@santotomas.cl (E.C.-M.); paulasalinasla@santotomas.cl (P.S.-L.); 5 Servicio de Neurología, Hospital Clínico Herminda Martin, Chillán 3810525, Chile; 6 Faculty of Economy and Business, Universidad Alberto Hurtado, Erasmo Escala 1835, Santiago 8340539, Chile; facrespo@gmail.com; 7 Faculty of Health and Social Sciences, Universidad de Las Américas, Sede Providencia, Manuel Montt 948, Santiago 7500975, Chile; nmunoz@udla.cl; 8 Centro de Investigación en Prevención y Cuidados de la Salud (+SALUD), Facultad de Salud, Universidad Santo Tomás, Santiago 8370003, Chile; dfreire2@santotomas.cl (D.F.-F.); ccarvallov@santotomas.cl (C.C.-V.)

**Keywords:** aged, mild cognitive impairment, memory, episodic, cognitive dysfunction, neuropsychological tests, early diagnosis, Mini-Mental State Examination, validation study, dementia

## Abstract

**Background**: Building on the validation of the Your Memory test for mild cognitive impairment in English speakers, this study adapted and validated the Memory Test for Mild Cognitive Impairment (TYM-MCI) for older Spanish-speaking adults, highlighting its potential utility for the early detection of amnestic mild cognitive impairment and cognitive profiles associated with increased risk of dementia. **Methods**: A total of 151 independently functioning adults aged 60 or older (Barthel Index 9–10) completed the TYM-MCI, the Addenbrooke’s Cognitive Examination-Revised (ACE-R-Ch), the Mini-Mental State Examination, and the original TYM. Analyses included ROC curves, correlation matrices, and principal component analysis (PCA). **Results**: The TYM-MCI exhibited strong psychometric properties (Cronbach’s α = 0.832; sensitivity = 81.7%; specificity = 47.8%). The optimal cut-off score was ≥24.5/30. Scores between 19 and 24.5 suggested probable mild cognitive impairment (MCI). **Conclusions**: The episodic memory components of this test are key cognitive features relevant to the modification and monitoring of early cognitive decline and are straightforward to administer. Notably, the TYM-MCI specifically assesses both visual and verbal episodic memory. It can be used alongside other assessments, such as the ACE-R or MMSE, to support the clinical evaluation of cognitive functioning in older adults. Clinically, it provides an early assessment and follow-up in individuals presenting with memory complaints, contributing to timely clinical decision-making in the context of cognitive decline.

## 1. Introduction

Memory is a fundamental cognitive function that supports an individual’s ability to adapt, autonomy, and quality of life, particularly in older adults [1,2]. Impairment in mnemonic processes is a key clinical indicator of neurocognitive decline and is frequently observed in conditions associated with an increased risk of dementia [3]. Neurocognitive disorders represent a heterogeneous group of conditions characterized by gradual, multifactorial brain dysfunction. They can be classified into several subtypes based on etiology, clinical features, disease progression, and associated conditions [4]. Within this framework, mild cognitive impairment (MCI), and particularly its amnestic subtype (aMCI), is commonly conceptualized as a clinical condition associated with an increased risk of progression to Alzheimer’s disease (AD), and other forms of dementia, while also allowing for alternative trajectories such as clinical stability or reversion to normal cognitive functioning [5,6,7]. Early cognitive changes in aMCI are frequently characterized by alterations in episodic memory (EM), a cognitive system responsible for the conscious recollection of autobiographical events situated in time and space, which is primarily in the hippocampus and distributed neocortical networks [8,9]. A growing body of evidence indicates that episodic memory impairment constitutes one of the most sensitive cognitive markers associated with an increased risk of cognitive decline in individuals with aMCI, particularly when deficits affect encoding and the delayed recall process [10,11]. However, it is important to note that episodic memory is not pathognomonic of AD, as similar profiles may be observed in other neurodegenerative, vascular, psychiatric, or potentially reversible conditions [12]. AD is a specific type of dementia, clinically characterized by progressive impairment in memory, language difficulties, executive functioning, and abstract reasoning [13,14]. Early identification of cognitive decline is therefore essential as it enables the timely implementation of therapeutic, psychosocial, and preventive interventions aimed at preserving functional independence and quality of life [15,16,17]. Several neuropsychological instruments have been validated to assess key cognitive domains and to support the early detection of MCI and dementia- related syndromes. In Chile, the Addenbrooke’s Cognitive Examination—revised (ACE-R-Chile) has demonstrated utility as a screening tool for global cognitive impairment [18,19]. Similarly, the Test Your Memory (TYM-S) and its version adapted for MCI (TYM-MCI) constitute brief and sensitive measures for the assessment of memory-related deficits in English-speaking populations. The TYM-MCI has shown advantages over traditional screening tools such as the MMSE, particularly in the evaluation of episodic memory performance [20,21]. Although advances in neuroimaging and biomarker research have enable the identification of biological markers associated with the prodromal stage of dementia [22,23], access to such techniques remains limited in many clinical settings. Consequently, brief, reliable, and culturally appropriate neuropsychological instruments continue to play a crucial role in the early clinical identification and monitoring of cognitive decline.

Compared with tools such as the TYM-S and the MMSE, which include limited verbal and visual memory components [22,23,24], the TYM-MCI incorporates a more detailed assessment of episodic memory, enhancing its sensitivity to amnestic cognitive profiles [25,26]. Importantly, the TYM-MCI has proven helpful in identifying clinically significant memory impairment in individuals who may still perform within normal ranges on global screening measures such as the MMSE or ACE-R [23]. Despite its demonstrated utility, evidence on the psychometric properties and clinical applicability of the TYM-MCI in Spanish-speaking populations remains scarce since Brown’s review in 2019 [20]. This gap is particularly relevant in countries such as Chile, where population aging is rapidly accelerating. In 2017, individuals aged 60 years and older accounted for 11.9% of the population, a proportion projected to increase to nearly 19% by 2035, alongside a sustained rise in life expectancy to around 80 years for men and 86 years for women [27,28,29,30]. In this demographic context, additional factors such as nutritional and physical status [31], hearing impairment [32], and perceived social status within reference groups [33] may further modulate cognitive aging trajectories. While neuroimaging techniques offer valuable diagnostic information, there is a pressing need for efficient, accessible, and low-cost cognitive screening strategies that facilitate early detection, timely intervention, and the prevention of unnecessary institutionalization [34].

Accordingly, the present study aims to adapt and validate the TYM-MCI for older Spanish-speaking adults, examining its psychometric properties and clinical utility for detecting episodic memory impairment associated with MCI. By doing so, this work seeks to contribute to the development of brief, culturally sensitive, evidence-based tools that support comprehensive, preventive geriatric care.

## 2. Materials and Methods

### 2.1. Design and Sample Population

This research employs a descriptive, non-experimental, cross-sectional design [35]. A total of 151 participants out of 239 were recruited through community-based purposive outreach programs and voluntary participation, resulting in a non-probabilistic convenience sample representative of active, community-dwelling older adults, following the approach of Brown et al. [20]. This sampling strategy was intentionally selected to support the initial psychometric validation of a cognitive screening instrument in an active, community-based population, rather than to estimate prevalence or ensure population-level representativeness. The sample had a 6% margin of error and a 95% confidence level. Given the non-probabilistic nature of the sampling, these parameters should be interpreted descriptively and do not imply population-level inference. Inclusion criteria were: being a native Spanish-speaker, living in the Metropolitan Region of Chile; aged 60 or older; without restrictions related to education or gender; possessing sufficient functional literacy to respond to the assessment tools; having maintained functional capacity (maximum score on the Barthel Index); and participating in community organizations for older adults. Exclusion criteria adhered to the provisions of Article 28 of Law No. 20,584 and Article 9, paragraph 5, of Law No. 21,331, both from the Republic of Chile, concerning human rights and mental health protections. Individuals with medical diagnoses or histories of psychiatric, neurological, or neurodegenerative disorders that hindered voluntary and conscious participation, as well as those with sensorimotor or physical limitations impairing their ability to perform assessment tasks, were excluded. The mild cognitive impairment (MCI) group was defined as those with scores between 81 and 91 on the ACE-R-Ch [19]. This classification was applied as an operational criterion for cognitive stratification within a screening context and does not constitute a formal clinical diagnosis. All procedures received approval from the Scientific Ethics Committee of the Universidad Mayor de Chile (protocol No. 0264, 13 July 2022, and Session 107, 13 September 2023), in compliance with the guidelines of the Declaration of Helsinki concerning research with human subjects [36].

### 2.2. Instrument

The assessments used include the Addenbrooke’s Cognitive Examination-Revised (ACE-R)—an instrument combining the MMSE that evaluates dementia with high sensitivity. It distinguishes Alzheimer’s disease (AD) from frontotemporal dementia (FTD), assessing temporal and spatial orientation, attention and concentration, memory, verbal fluency, language, and visuospatial skills. The cut-off score for mild cognitive impairment (MCI) is 81–91 out of 100 points. Scores below 81 suggest early AD. Reliability indicators are: reliability = 0.918; sensitivity = 0.917; and specificity = 0.93316 [18,19].

Test Your Memory (TYM-S): A brief assessment covering cognitive domains such as temporal-spatial orientation, autobiographical, semantic, and delayed memory, as well as attention, calculation, phonological and categorical fluency, and visuo-perceptual and visuo-constructive skills. The cut-off for MCI is ≤42/50, and for dementia it is <39. It demonstrates acceptable reliability (α = 0.776), sensitivity (0.857), and specificity (0.690) [22,24].

TYM-MCI (Test Your Memory for Mild Cognitive Impairment): A specialized version designed to detect amnestic MCI and Alzheimer’s disease, focusing on episodic memory (verbal and visual delayed recall), comprehension, delayed recall, recognition, and drawing recall. The cutoff point is ≤13/30. Reported sensitivity is 0.67, with a specificity of 0.66 [20,21].

Mini-Mental State Examination (MMSE): A screening tool that assesses basic cognitive functions such as orientation, memory, attention, calculation, language, and spatial skills [37,38].

Barthel Index: An instrument measuring functional independence in basic daily activities, serving as a supplementary indicator of cognitive integrity and functional autonomy, evaluated in nutritional contexts [39].

### 2.3. Procedure

The TYM-MCI [21,29] was translated into Spanish using the translation-back-translation method to ensure semantic and conceptual equivalence. It was then reviewed by an expert panel comprising three psychologists experienced in neuropsychology and one professional translator. A rating matrix based on the Aiken V index and a Likert scale was used to assess the relevance, clarity, and consistency of the items within the cognitive domain, with an acceptance threshold of 95% [40]. Testing took place in a quiet 3 × 3 m room provided by the Communal Union of Older Adults (UCAM) in Melipilla city, Chile. Participants first signed the informed consent and completed the Barthel assessment. Subsequently, the ACE-R-Ch with MMSE was administered, followed by the TYM and TYM-MCI tests. Researchers supported the older adults during testing, who completed minimal self-administered tasks. In a follow-up session, results were discussed along with suggestions for improving performance or maintaining cognitive strengths. The order of test administration followed a pragmatic clinical sequence, progressing from functional assessment to global cognitive screening and subsequently to domain-specific memory evaluation.

### 2.4. Statistical Analysis

The ROC curve, correlation analysis, and Principal Component Analysis (PCA) were performed using R software version 4.2.1. This process allows for the identification of cutoff points for cognitive impairment by calculating sensitivity and specificity for the TYM-MCI [41]. PCA reduces data complexity, facilitating the interpretation of results by revealing underlying variables [42]. The use of parametric statistics depended on the normality analysis, which was conducted using the Kolmogorov–Smirnov (K-S) test. Coefficients for homogeneity and reliability were calculated with Cronbach’s alpha. The cut-off score for estimating cognitive impairment was determined via the ROC curve based on sensitivity and specificity data [41]. Data were analyzed using IBM SPSS version 29.0 and GraphPad Prism 6.0 for descriptive purposes. In summary, the following procedures were applied: a normality test (Kolmogorov–Smirnov) to guide the use of parametric procedures, Cronbach’s alpha to assess internal consistency, ROC curves to identify optimal cutoff points based on diagnostic sensitivity and specificity [41] and PCA to reduce data dimensions and explore latent variables related to cognitive domains, aiding the structural interpretation of the test [42,43].

## 3. Results

The principal quantitative findings obtained in the validation and cultural adaptation of the TYM-MCI in older Spanish-speaking adults are presented.

### 3.1. Descriptive Data and Sample Distribution

The Kolmogorov–Smirnov test confirmed normal data distribution for all test measures among the 151 community-dwelling older adults evaluated. Mean scores were ACE-R-Ch = 91.82 (SD = 4.13; *p* = 0.15), MMSE = 28.35 (SD = 2.14; *p* = 0.32), TYM = 44.19 (SD = 2.69; *p* = 0.15), and TYM-MCI = 23.48 (SD = 2.82; *p* = 0.0098). Participants’ mean age was 72.9 years (SD = 7.7), with a predominance of women. 30.1% reported having a technical or university education, with average educational level of 11.21 (SD = 2.76) and participated regularly in cognitively stimulating activities, such as reading and community workshops.

### 3.2. Internal Consistency, Prevalence of Impairment, and ROC Curve of the TYM-MCI

**Figure 1:**
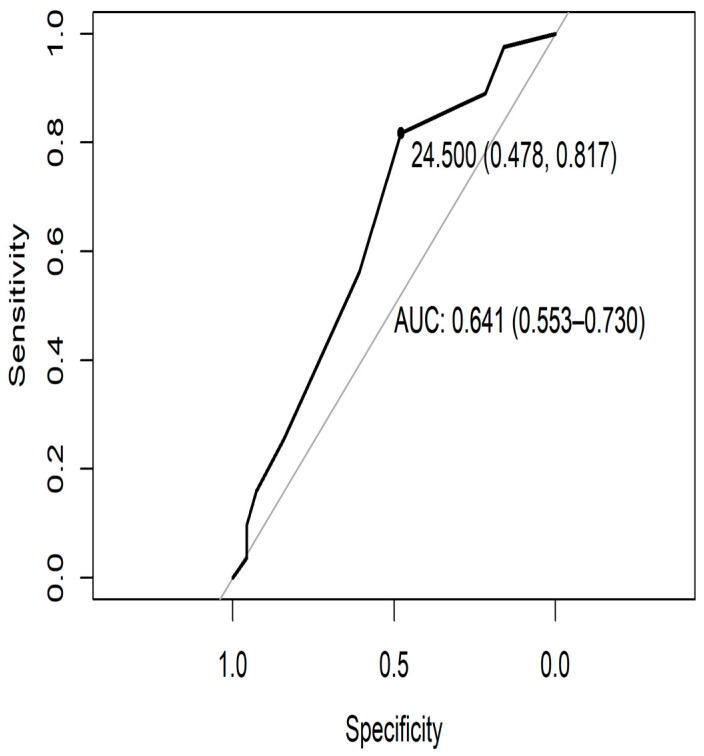
ROC curve for TYM-MCI with a cut-off point and area under the curve (AUC).

The TYM-MCI demonstrated high internal consistency, with a Cronbach’s alpha of 0.832. ROC curve analysis identified an optimal cutoff score of 24.5 points, with a sensitivity of 81.7% and a specificity of 47.8%. A total of 239 older adults were evaluated, of whom 151 met the inclusion criteria. Eighty-nine (58.9%) individuals were classified as having amnestic MCI, as mentioned. The remaining individuals were within the standard neuronormative range, according to the TYM-MCI cutoff point. accounting for 58.9% of the sample. The area under the curve (AUC) was 0.641, indicating moderate discriminatory ability (Figure 1 and Table 1).

### 3.3. Correlation Analysis Between Cognitive Tests

Pearson’s correlation analysis between the different instruments showed that the TYM-MCI has a weak to moderate correlation with the other tests. The strongest Pearson correlation was with the TYM (r = 0.348), followed by the ACE-R (r = 0.254), and then the MMSE (r = 0.186). All correlations were significant at *p* < 0.01. These findings suggest that the TYM-MCI assesses somewhat different aspects of cognitive functioning, supporting its additional value in diagnosing MCI. Additionally, there is a moderate correlation between the ACE-R and MMSE tests (r = 0.54) and ACE-R TYM (r = 0.58) and a low correlation between MMSE and TYM (r = 0.32).

### 3.4. Sensitivity and Specificity by Cognitive Domains

The analysis of diagnostic accuracy indicates that for the episodic memory domain, the TYM-MCI achieved a specificity of 83.1% and an accuracy of 67.5%, although its sensitivity was low at 12.1%. In contrast, the ACE-R demonstrated a more balanced ratio between sensitivity (33.3%) and specificity (80.5%). When assessing global cognitive impairment, the ACE-R achieved an accuracy of 97.4%, with high sensitivity (94.2%) and specificity (100%). The TYM-MCI also showed high specificity (81.7%) but had an intermediate sensitivity (47.8%) (see Table 2).

### 3.5. Principal Components Analysis (PCA): State Variables

The PCA performed for each of the cognitive tests is shown in the following Table 3 and Table 4, along with the corresponding figures, which will be indicated.

### 3.6. PCA of ACE-R

**Figure 2:**
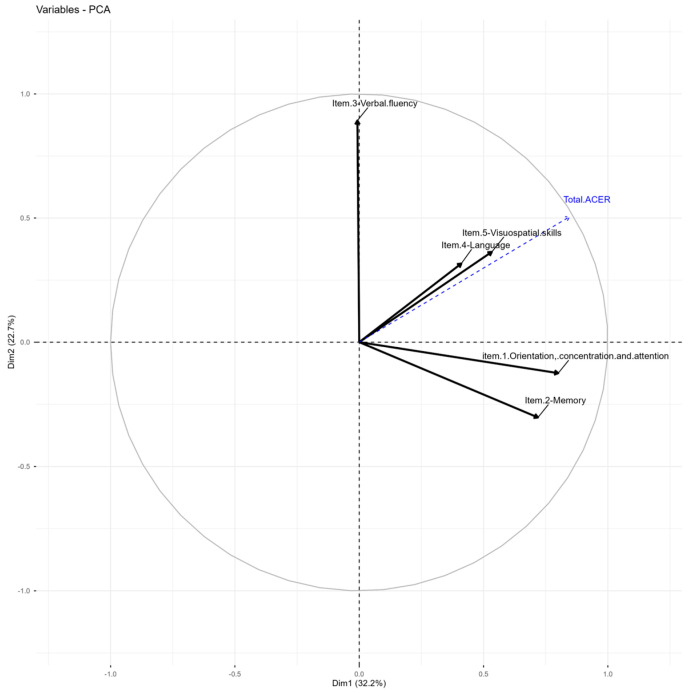
PCA Graph of variables ACE-R test. Variables PCA graph: The first two implicit dimensions removed from the PCA with high-correlation ACE-R data are considered.

Three components explain 76.18% of the total variance (Table 3). Orientation and memory cluster in the first dimension, showing a high correlation with the total test score (r = 0.85). Verbal fluency clusters in the second dimension as an independent variable, and language associates with the visuospatial component in the third dimension, although in different directions (Table 4 and Figure 2). The suitability of the five ACE-R items, measured using the Kaiser-Meyer-Olkin (KMO) index, indicates acceptable scores for item (1) Orientation, Concentration, and Attention (0.52); item (2) Memory (0.52) and item (4) Language (0.50). The items with insufficient suitability are: item (3) Verbal Fluency (0.42) and item (5) Visuospatial Skills (0.49). Bartlett’s Test of Sphericity shows a significance R of <0.01, indicating that the correlation matrix is not an identity matrix.

### 3.7. PCA of the MMSE

**Figure 3:**
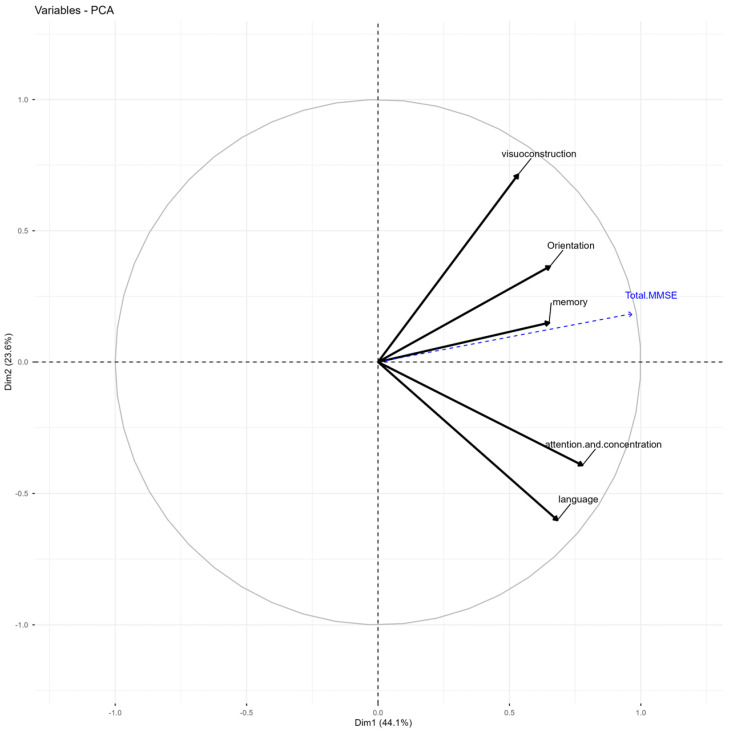
Graph of variables MMSE test. Variables PCA graph: Items of MMSE work together as a group, not independently.

Three components accounted for 83.68% of the variance (Table 3). The overall test showed a high correlation with the first dimension (r = 0.97). In the second dimension, the language subtest exhibited a strong negative correlation. In contrast, visuospatial construction showed a positive but low association with the total MMSE (Table 4 and Figure 3). The five MMSE items had acceptable KMO values, as indicated by Item 1. Orientation = 0.66; Item 2. Attention and Concentration = 0.61; Item 3. Memory = 0.68; Item 4. Language = 0.56; and Item 5. Visuoconstruction = 0.55. Bartlett’s test of sphericity showed a significance level less than R = 0.01, demonstrating that the correlation matrix is not an identity matrix.

### 3.8. PCA of the TYM-S

**Figure 4:**
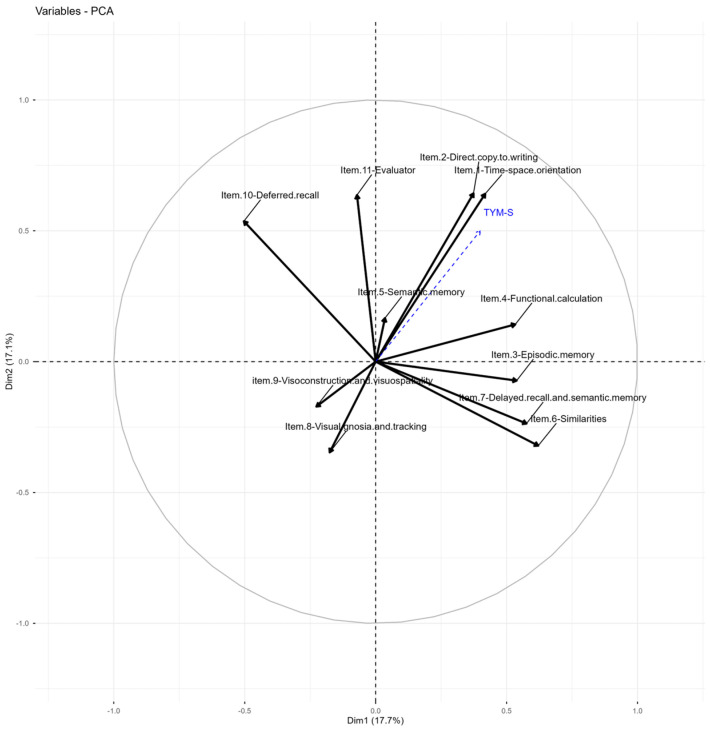
PCA Graph of variables TYM-S test. Variables PCA graph: Items of TYM test work with structure disperse of variables. Compare with Table 4.

Five components explained 67,65% of the variance (see Table 3). The structure of the TYM-S is dispersed, with a low correlation between the total score and the component factors. Dimension 1 strongly groups items related to lexical similarities (item 6), functional calculation (item 4), delayed recall and semantic memory (item 7), and episodic memory (item 3). These results suggest the emergence of a component related to complex verbal reasoning and active memory processes, possibly linked to verbal cognitive reserve and the executive integration of verbal-semantic content. Dimension 2 groups items on time-spatial orientation (item 1), direct copying (item 2), and the evaluator item (item 11), suggesting a second component more closely linked to simple visual-constructional skills and attentional monitoring or basic execution tasks. Dimension 3 is characterized by a strong positive loading on the semantic memory item (item 5) and significant negative loadings on visual agnosia and tracking (item 8), which could represent an opposite gradient between conceptual retrieval and visual perceptual identification, suggesting a differentiation between visual and semantic processing. Dimension 4 shows a significant loading on visuospatial skills, specifically on the visuospatial skills item (item 9), indicating the emergence of a specific factor linked to complex visuospatial skills that is relatively independent of the other functions. Dimension 5 is notable for its high loadings on functional calculation (item 4) and semantic memory (item 5), components that can be associated with acquired cognitive reserve (arithmetic fluency and cultural knowledge). Dimensions 6 and 7, although with less explanatory weight, contain significant correlations with delayed episodic memory (item 3) and visual components such as agnosia and visuospatial construction, suggesting a diversity of individual strategies for visual and verbal recall. The total TYM score correlates moderately with the first five dimensions (higher in Dim. 2 and Dim. 1), confirming a multifactorial structure of the test, with orientation, similarity, memory, and calculation tasks contributing most to overall performance (Table 4 and Figure 4).

The KMO measures acceptable are Item 1—Time-Space Orientation (0.47), Item 2—Direct Copy to Writing (0.47), Item 3—Episodic Memory (0.66), Item 4—Functional Calculation (0.64), Item 5—Semantic Memory (0.37), Item 6—Similarities (0.62), Item 7—Delayed Recall and Semantic Memory (0.42), Item 8—Visual Gnosis and Tracking (0.57), Item 9—Visuoconstruction and Visuospatial Skills (0.47), Item 10—Deferred Recall (0.64), and Item 11—Evaluator (0.67). The KMO is unacceptable for Semantic Memory, Delayed Recall, and Visuospatial Memory, as well as Visuospatial skills; for the other variables, it is mediocre. Bartlett’s Test of Sphericity has a significance level of less than 0.01, indicating that the correlation matrix is not an identity matrix.

Table 3 shows the correlation between the variables and the eigenvectors, with the total TYM test score as the dependent variable. From this, it is clear that the total TYM score has a very low correlation with the third dimension onward, which shows a high positive correlation with the item Semantic Memory and a high negative correlation with the items Visual Gnosia and Tracking. The correlation with four dimensions is nearly zero, and these four dimensions have a strong correlation with item nine of Visuo-construction. The correlation of TYM with the fifth dimension is low (0.31), and this dimension is highly correlated with Item 4—Functional Calculation and Item 5—Semantic Memory.

### 3.9. PCA of the TYM-MCI

**Figure 5:**
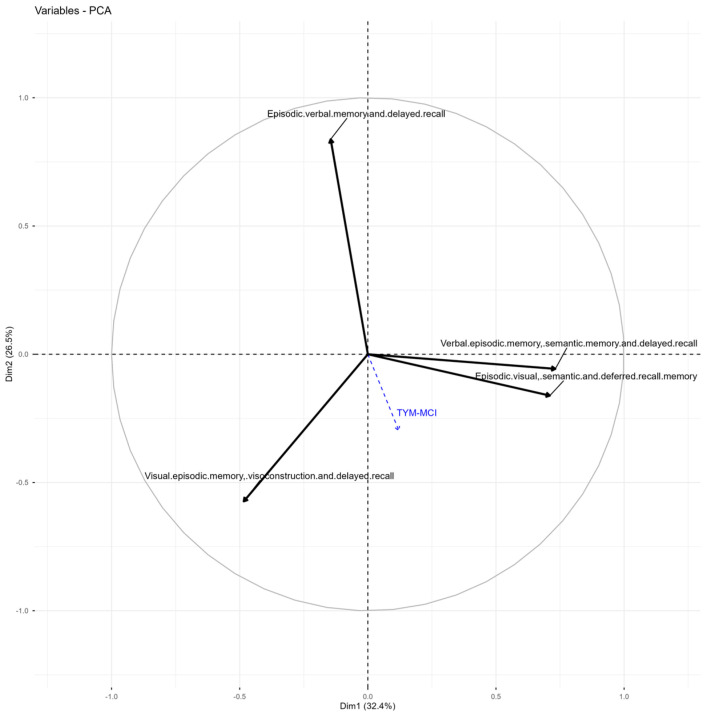
Graph of variables TYM-MCI test. Variables PCA graph: Items of TYM-MCI test work independently.

Three components explained 80.4% of the variance (Table 3). The total test score was strongly correlated with the third dimension (r = 0.93), where visual and verbal episodic memory converge, giving these items structural significance compared to other instruments. Specifically, Dimension 1 exhibits strong positive correlations with the verbal and semantic components, particularly with items 3 and 4. Dimension 2 is strongly related to verbal episodic and delayed recall memory, indicating an independent axis of verbal processing. Dimension 3 is closely aligned with the total test score (0.933) and loads significantly on both verbal and visual components, suggesting this dimension represents the integrative factor of overall episodic memory performance. Dimension 4 presents opposite correlations between visual and semantic items, which could indicate a tension between visual versus verbal/semantic encoding (Table 4 and Figure 5).

The KMO values are moderate for all variables: 0.55 for Visual Episodic Memory, Visuoconstruction, and Delayed Recall; 0.55 for Episodic Verbal Memory and Delayed Recall; 0.48 for Verbal Episodic Memory, Semantic Memory, and Delayed Recall; and 0.54 for Episodic Visual, Semantic, and Deferred Recall Memory. Bartlett’s Test of Sphericity has a significance of less than 0.01, indicating that the correlation matrix is not an identity matrix. TYM-MCI scores, which shows high positive or negative correlations with some subtests. All of this is new compared to previous tests, which had a weaker relationship with visual items.

## 4. Discussion

### 4.1. Global Analyses

The overall results of the TYM-MCI test confirm its effectiveness in assessing episodic memory (EM) performance, as initially reported by Brown et al. (2014) [26]. Its high internal consistency and reliability, compared to other screening tools, likely stem from its ability to focus on a specific cognitive domain using criteria that help detect mental impairment, as shown by ROC curve analysis—a standard method in such validation studies [41]. From a preventive and public health perspective, prioritizing sensitivity is particularly relevant in early screening contexts, as it minimizes the risk of overlooking individuals with clinically meaningful cognitive changes who may benefit from further assessment and follow-up.

Visual and verbal episodic memory assessments have been consistently associated with aMCI and with an increased risk of future cognitive decline, rather than constituting diagnostic or etiological markers of AD per se [44,45,46]. Along with limitations in daily living activities, these are essential criteria for early dementia diagnosis [47]. In addition, the Barthel Index scores in this study ranged from 9 to 10 in all participants, indicating preserved functionality—a critical factor in ruling out early functional decline [48]. Such indicators are recommended in clinical practice, as they affect occupational, social, and family performance, consistent with the MMSE’s validation in Chile using the Pfeffer questionnaire [38]. Early cognitive signs include deficits in learning and retaining new information, disorientation in time and space, and significant changes in autobiographical memory, which aid in the clinical identification of aMCI prior to overt dementia, without assuming a specific underlying etiology [45,49]. The specific EM contexts measured by the TYM-MCI differ from those assessed by other tools, improving the accuracy of neuropsychological evaluation [50,51,52].

The optimal cut-off (≥24.5) achieved high sensitivity (81.7%) but moderate specificity (47.8%), reflecting a strong capacity to identify individuals with mild cognitive impairment while minimizing false negatives—an important consideration in preventive and community-based settings. The lower specificity and consequent increase in false-positive classifications are consistent with the explicit design of the TYM-MCI to detect subtle episodic memory (EM) changes, a cognitive domain particularly vulnerable in the earliest stages of decline [25,49,50]. As a result, the instrument may identify preclinical or subthreshold alterations that are not captured by broader cognitive screening tools such as the MMSE or ACE-R. This effect may be further accentuated in community-dwelling, highly functional, and relatively well-educated samples, in which subtle episodic memory deficits may already be present despite preserved global cognition [53,54]. From a clinical perspective, these false-positive results should not be interpreted as diagnostic errors but rather as indicators warranting further assessment. Positive screening findings on the TYM-MCI should prompt comprehensive neuropsychological evaluation and, when appropriate, additional clinical, biomarker, or neuroimaging investigations. Accordingly, alternative cut-off thresholds may be considered depending on the intended use of the instrument, with higher sensitivity thresholds favored for early detection and lower thresholds reserved for confirmatory or research-oriented contexts.

Educational attainment is known to influence performance on cognitive screening instruments in older adults. Population-based studies have shown that lower levels of formal education are associated with lower baseline scores on global tests such as the Mini-Mental State Examination, increasing the risk of false-positive classifications in cognitively normal individuals [55]. More recent evidence confirms that this educational effect remains relevant in contemporary clinical settings and highlights the need for cautious interpretation of screening results in populations with heterogeneous educational backgrounds [56]. At the same time, research suggests that cognitive measures focusing on episodic memory—particularly those incorporating visual components—may be relatively less dependent on education than global screening instruments, although education-related effects are not completely eliminated [57]. Accordingly, the proposed cut-off may not be directly transferable to populations with lower educational levels, rural backgrounds, or greater clinical impairment without further context-specific validation. From a public health perspective, prioritizing sensitivity reduces the risk of missing at-risk cases, even if secondary confirmatory assessments are needed. The TYM-MCI thus serves as an ideal initial screening tool to be supplemented by more comprehensive neuropsychological batteries. This approach aligns with the ecological variability of EM, observable even in populations without obvious impairment and cognitive reserve [54,58].

The difference between the present current cut-off (24.5) and that proposed by Brown et al. [20,21], (13.1) is due to sociodemographic and cognitive differences in the samples. The original study included patients from the neurology clinic with a high pretest probability of impairment. In contrast, our cohort consisted of functioning, autonomous older adults (Barthel 9–10) engaged in community activities in Chile, with medium- to high-educational and a younger mean age (72 years). Although primarily focused on amnestic profiles, the TYM-MCI may reveal atypical visuospatial or language-related performance patterns that prompt further evaluation for non-amnestic trajectories of cognitive decline, without implying etiological differentiation [7,15,46]. In high-reserve populations, baseline performance is higher, requiring stricter cut-offs to detect significant decline [59]. Thus, a higher cut-off is expected in active, non-clinical samples, reflecting appropriate local adaptation [60]. In Chile and Latin America, evaluation of EM remains limited and under-validated despite growing research [61]. Screening tools validated in similar domains include the ACE-R-Ch [19], the MMSE [38], the TYM [24], and the IFS [62]. Correlation analyses show significant associations between most instruments, except for the TYM-MCI, whose lower correlation suggests it measures distinct domains. This reinforces its specificity for EM, particularly in distinguishing amnestic pattern of cognitive impairment from other cognitive profiles of cognitive impairment [20,21].

The TYM-MCI also provides specific features to evaluate verbal and visual episodic memory—key indicators for early clinical identification of amnestic cognitive impairment—through tasks like direct copying and delayed recall [21]. This information provides crucial diagnostic clues and helps distinguish different types of dementia when combined with assessments of daily living skills [4,12,17]. Furthermore, by evaluating amnestic symptoms that affect daily activities, the TYM-MCI conducts assessments under standardized conditions that reflect functional and predictive relationships of ecological validity [63,64]. Clinically, performance tends to decline in routine tasks within family, work, and social settings, which correlates with hippocampal white matter development and organization [65]. Complex event patterns activate hippocampal areas, with familiarity rooted in the perirhinal cortex, which is sensitive to repetition [10]. Palliative strategies, such as distraction-based activities, may stimulate cognition by leveraging episodic memory [66].

### 4.2. Analysis of the Relationship Between Cognitive Tests Regarding Episodic Memory

Exploratory principal components analysis (PCA) of each test allowed us to evaluate the contribution of TYM-MCI to the assessment of mental impairment. Since each item influences the final test score, this impact is readily observable. We used the individual item scores to determine the overall total score as a supplemental quantitative measure. This approach enables us to analyze the correlations between the original items and their components, and to assess how these variables influence the final score within the observed sample. The importance of the variables in each item varies with the final score, which will be examined more thoroughly using PCA [42].

The correlation of the ACE-R eigenvector variables suggests that the orientation and memory items do not correspond to distinct variables, given their high correlations and the total test score. Therefore, the individual being assessed might be relying on the same cognitive process for these items. The high correlation of the verbal influence item in dimension 2 with other components demonstrates its independence from different items. The language item centers on the visuospatial item in dimension 3, indicating a strong likelihood that performance on the visuospatial task depends on language skills. Understanding instructions may be a key factor in the visuospatial test, which could explain why the clarity of the evaluator’s expression influences performance on the subtest, as evidenced by the correlation between these variables (see Figure 2 and Table 2). Evidence indicates that language affects cognitive reserve, which may be reflected in optimal mental performance, explaining this relationship [58,67,68].

Regarding the MMSE test, the first component (Dim. 1) heavily groups all the classic items of the test, with exceptionally high loadings on the subtests. This test suggests that the first dimension represents a global cognitive function, or general factor (g), that explains a significant portion of MMSE performance. The high correlation with the total MMSE (0.97) supports the view that this test mainly functions as a unidimensional measure of overall cognitive performance (as supported by previous studies [69]). Regarding episodic memory representation, although it shares variance with overall performance, this cognitive process is not confined to dimension one but spans dimensions 1 and 3, distinguishing it from other mental functions. However, the MMSE is limited to immediate word recall and delayed evocation without greater complexity [22,37], and episodic memory assessed here does not significantly influence the overall test score [70]. Conversely, a dissociation between visuospatial and verbal functions has been identified due to contradictory loadings in the PCA. The MMSE is only moderately sensitive and specific for episodic memory, limiting its utility in evaluating older adults. Instead, it is designed as a broad screening tool for cognitive decline, which is why it should be supplemented with the TYM-MCI [21] or the ACE-R [19].

Furthermore, the PCA results for the TYM uncover a complex, partially multidimensional structure, consistent with this instrument being a brief, heterogeneous screening test that includes verbal, visuospatial, and memory components [20,24]. The presence of multiple dimensions suggests that the TYM does not measure a single cognitive construct but rather a set of cognitive subfunctions. This pattern aligns with previous findings indicating that screening tests capture different types of cognitive decline, depending on cognitive reserves, educational background, or neuropsychological aging trajectories [54,70].

However, identifying a separate visuospatial construction component enhances the clinical utility of the TYM in detecting specific deficits, especially in non-amnestic dementias (e.g., frontotemporal dementia or non-amnestic mild cognitive impairment). Additionally, the combination of verbal and visual tasks across domains may reflect the various compensatory strategies older adults employ. Finally, the association of semantic memory with both executive functions and perceptual and visuospatial components emphasizes its role as an integrative link between knowledge and retrieval processes, which is vital during the early stages of mild cognitive impairment [7,15,46]. Importantly, the present findings should be interpreted within a clinical–cognitive framework. In the absence of biomarker or neuroimaging confirmation, the TYM-MCI does not aim to establish an etiological diagnosis, but rather to identify amnestic cognitive profiles associated with an increased risk of cognitive decline. Although primarily focused on amnestic profiles, the TYM-MCI may reveal atypical visuospatial or language-related performance patterns that prompt further evaluation for non-amnestic trajectories of cognitive decline, without implying etiological differentiation.

### 4.3. Does the TYM-MCI Effectively Assess Episodic Memory Compared to Other Tests?

In summary, it is possible to demonstrate:

(a) Concerning the consistency of the factor structure with mixed memory systems, the results show that the tasks included in the TYM-MCI, although mainly designed to evaluate episodic memory, activate multiple systems (verbal, visual, semantic episodic memory, and consolidation processes). This consistency is expected, as episodic memory rarely occurs in isolation and relies on neurocognitive networks that integrate semantics, attention, and multisensory encoding [7,8].
(b) An empirical finding of a general episodic memory factor appears in Dimension 3, which shows a high correlation with the total TYM-MCI score (r = 0.933). This high correlation confirms that the test has internal structural coherence, with items converging on a single factor. This consistency indicates that the test effectively synthesizes a strong representation of episodic performance (both verbal and visual) within its total score [22], supporting its internal validity.
(c) Furthermore, processing differences between visual and verbal modalities serve as a functional dissociation, as Dimensions 1 and 2 suggest a relative separation between these modalities. Dimension 2, which focuses on verbal recall, is the most independent, while the visual dimension is spread across other dimensions. This variability could be due to individual differences in encoding strategies (dual coding) or to selective impairments in patients with amnestic mild cognitive impairment (aMCI), who often show reduced verbal episodic memory rather than visual memory [16,71].
(d) The compensatory gradient, indicated by the opposite signs between visual and verbal items in Dimension 4, may reflect individual compensatory strategies: individuals with difficulties in visual tasks may compensate via the semantic-verbal domain, and vice versa. This finding is clinically significant as it helps outline different cognitive pathways in mild impairment, with implications for personalized rehabilitation [17]. Factor analysis of the TYM-MCI indicates that this tool effectively captures a core aspect of episodic memory, particularly given its combined verbal, visual, and delayed recall components. The high load of the total score on the third dimension, along with the observed dissociations between sensory modalities, suggests that the TYM-MCI is a brief yet well-structured test for detecting mild cognitive impairment, with a particular focus on episodic memory, and is especially sensitive to early and mixed memory issues [20].
(e) Recent evidence supports integrating brief cognitive screening tools with noninvasive biomarker approaches in the early stages of cognitive decline. In particular, studies have shown that plasma tau and cerebrospinal fluid biomarkers, among others, can improve early risk stratification and help identify individuals who might benefit from further assessment within disease-modifying clinical trials when used in structured diagnostic frameworks [72,73]. In this context, the TYM-MCI can serve as an initial cognitive filter, identifying amnesic profiles that warrant further biomarker-based assessment, thereby increasing the translational applicability of current findings without exceeding the instrument’s intended scope.

## 5. Conclusions

### 5.1. General Conclusions

The results of the principal components analysis (PCA) and the comparison with other cognitive screening tools confirm that the TYM-MCI is a sensitive and specific instrument for detecting amnestic-type mild cognitive impairment. It also meets reliability and internal consistency criteria, with a cutoff score of 24.5 in older adults with cognitive reserve. Unlike other general assessments, such as the MMSE or the ACE-R, the TYM-MCI exhibits a clear factor structure focused on episodic memory, encompassing visual, verbal, and semantic components, with a high factor loading on a primary dimension that consolidates this performance. This structural coherence enhances its internal validity as a specialized test.

Furthermore, the functional separation between the visual and verbal modalities suggests that the TYM-MCI can identify compensatory patterns or different cognitive profiles, increasing its clinical usefulness in early clinical identification and planning personalized interventions. In this regard, the TYM-MCI not only effectively distinguishes cases of amnestic-type mild cognitive impairment but also provides indicators of cognitive reserve, encoding strategies, and monitoring of cognitive trajectories over time, making it a valuable tool for a comprehensive approach to cognitive deficits in older adults. Therefore, it is recommended to combine it with broader and functional assessments in neuropsychological evaluation protocols for older adults, especially during the early stages of cognitive decline when episodic memory is a key clinical marker. Overall, the psychometric, clinical, and contextual results confirm that the TYM-MCI is a valid, reliable, and culturally and educationally appropriate tool for older Spanish-speaking adults, with a clear focus on episodic memory as an early indicator of cognitive decline. It is curious that, after the publication of Brown et al. (2019) [20], there are no reports of TYM-MCI use in the Spanish-speaking population, which makes this study relevant, in addition to what has been indicated above.

### 5.2. Strengths and Limitations of the Study

This study has several strengths. It represents one of the few validations of the TYM-MCI conducted specifically in older Spanish-speaking adults, a population for which culturally adapted cognitive screening tools remain limited. The use of a functionally independent, community-based sample with medium to high educational levels enhances external validity and reflects performance in active aging contexts. In addition, the application of advanced statistical approaches, including principal component analysis, provides robust psychometric evidence supporting the specific contribution of episodic memory components to the overall test performance. The TYM-MCI also demonstrated good internal consistency (α = 0.832) and high sensitivity, supporting its utility as a first-line screening tool for early cognitive decline.

Several limitations should also be acknowledged. The moderate-to-low specificity (47.8%) indicates a risk of false-positive classifications, particularly among highly educated individuals with greater cognitive reserve, underscoring the need for confirmatory assessment using complementary neuropsychological measures. The cross-sectional design precludes conclusions regarding predictive validity, highlighting the importance of longitudinal studies to evaluate the ability of the TYM-MCI to monitor cognitive trajectories over time. Furthermore, although the sample is representative of active older adults, it may not adequately reflect rural, low-literacy, or socioeconomically vulnerable populations, limiting generalizability.

Finally, the absence of biomarker or neuroimaging data restricts etiological inference. Accordingly, the TYM-MCI should be interpreted as an initial cognitive screening instrument within a multistep diagnostic framework rather than as a stand-alone tool for etiological classification or clinical trial selection. Future studies incorporating biomarker data and more diverse educational backgrounds will be essential to further establish its predictive and translational value.

## Figures and Tables

**Table 1:** Specificity and sensitivity values for the TYM-MCI Test, as calculated by the ROC curve.

Score *	Sensitivity *	Specificity *
27.5	0.976	0.159
26.5	0.890	0.217
25.5	0.866	0.304
24.5	0.817	0.478
23.5	0.561	0.609
22.5	0.256	0.841
21.5	0.159	0.928
20.5	0.098	0.957
19.5	0.073	0.957
18.5	0.037	0.957

* Positive if greater than or equal to the value.

**Table 2:** Accuracy, Sensitivity and Specificity of Cognitive Test.

Episodic Memory					Global Cognitive				
	ACER	MMSE	TYM	TYM-MCI		ACER	MMSE	TYM	TYM-MCI
Accuracy	0.702	0.238	0.623	0.675	Accuracy	0.974	0.729	0.828	0.662
Sensitivity	0.333	0.879	0.333	0.121	Sensitivity	0.942	0.841	0.942	0.478
Specificity	0.805	0.059	0.703	0.831	Specificity	1.00	0.634	0.732	0.817

**Table 3:** Eigenvector components of each test.

Components of Test	Eigenvalue	% Variance	Cumulative % Variance
comp 1 ACE R	1.61	32.21	32.21
comp 2 ACE R	1.14	22.74	54.95
comp 3 ACE R	1.06	21.23	76.18
comp 4 ACE R	0.674	13.49	89.66
comp 5 ACE R	0.512	10.34	100.00
comp 1 MMSE	2.21	44.12	44.12
comp 2 MMSE	1.18	23.63	67.76
comp 3 MMSE	0.80	15.93	83.69
comp 4 MMSE	0.47	9.41	93.09
comp 5 MMSE	0.35	6.91	100.00
comp 1 TYM	1.95	17.71	17.71
comp 2 TYM	1.88	17.09	34.80
comp 3 TYM	1.46	13.22	48.02
comp 4 TYM	1.12	10.18	58.21
comp 5 TYM	1.04	9.44	67.65
comp 6 TYM	0.76	6.89	74.54
comp 7 TYM	0.68	6.15	80.69
comp 8 TYM	0.65	5.90	86.58
comp 9 TYM	0.61	5.48	92.07
comp 10 TYM	0.56	5.09	97.16
comp 11 TYM	0.31	2.84	100.00
comp 1 TYM-MCI	1.295	32.384	32.384
comp 2 TYM-MCI	1.061	26.517	58.901
comp 3 TYM-MCI	0.860	21.499	80.401
comp 4 TYM-MCI	0.784	19.599	100.000

**Table 4:** Correlations variables eigenvectors cognitive test. Each test projected as the dependent variable.

Subitem	Dim. 1	Dim. 2	Dim. 3	Dim. 4	Dim. 5	Dim. 6	Dim. 7
Orientation ACE-R	0.80	−0.13	−0.26	0.16	−0.50	X	X
Memory ACE-R	0.72	−0.30	0.32	0.35	0.40	X	X
Verbal fluency ACE-R	−0.007	0.89	0.06	0.45	−0.01	X	X
Language ACE-R	0.41	0.32	0.72	−0.44	−0.11	X	X
Visuospatial skills ACE-R	0.53	0.36	−0.60	−0.36	0.30	X	X
Total ACE-R	0.85	0.51	0.03	0.14	0.09	X	X
Orientation MMSE	0.65	0.36	−0.53	−0.38	0.09	X	X
Attention and concentration MMSE	0.78	−0.39	−0.22	0.18	−0.40	X	X
Memory MMSE	0.65	0.15	0.67	−0.30	−0.10	X	X
Language MMSE	0.68	−0.60	0.07	0.11	0.40	X	X
Visuospatial construction MMSE	0.53	0.71	0.07	0.44	0.08	X	X
Total MMSE	0.97	0.19	0.054	−0.15	0.002	X	X
Time-space orientation TYM	0.42	0.64	−0.31	0.15	0.21	−0.16	−0.12
Direct copy to writing TYM	0.37	0.64	−0.24	0.18	−0.27	0.11	0.40
Episodic memory TYM	0.54	−0.07	0.15	0.49	−0.17	0.42	−0.48
Functional calculation TYM	0.53	0.14	−0.08	0.03	0.67	0.04	0.15
Semantic memory TYM	0.04	0.17	0.68	−0.27	0.46	0.17	−0.06
Similarities TYM	0.62	−0.32	0.04	−0.28	−0.07	−0.41	−0.12
Delayed recall and semantic memory TYM	0.58	−0.24	0.39	−0.29	−0.29	0.30	0.36
Visual agnosia and tracking TYM	−0.18	−0.35	−0.60	−0.20	0.30	0.44	0.03
Visuoconstruction and visuospatial skills TYM	−0.23	−0.17	0.46	0.66	0.20	−0.13	0.25
Deferred recall TYM	−0.50	0.54	0.14	−0.07	−0.08	0.19	0.005
Evaluator TYM	−0.07	0.64	0.26	−0.33	−0.08	−0.01	−0.20
Total TYM	0.40	0.50	0.21	−0.004	0.31	0.26	0.06
Visual episodic memory + visoconstruction + delayed recall	−0.48	−0.57	0.65	0.10	X	X	X
Episodic verbal memory + delayed recall	−0.15	0.84	0.53	0.001	X	X	X
Verbal episodic memory + semantic + delayed recall	0.73	−0.06	0.21	0.65	X	X	X
Episodic visual + semantic + deferred recall	0.71	−0.16	0.34	−0.60	X	X	X
Total TYM-MCI	0.12	−0.30	0.93	0.16	X	X	X

## Data Availability

The original contributions presented in this study are included in the article. For more information, please contact the corresponding author.
